# Role and application of three-dimensional transthoracic echocardiography in the assessment of left and right ventricular volumes and ejection fraction: a UK nationwide survey

**DOI:** 10.1186/s44156-024-00044-1

**Published:** 2024-04-03

**Authors:** Liam Corbett, Patrick O’Driscoll, Maria Paton, David Oxborough, Elena Surkova

**Affiliations:** 1 Liverpool Heart and Chest Hospital NHS Foundation Trust, Liverpool, UK; 2 University of Leeds, Leeds, UK; 3 Research Institute of Sports and Exercise Science and Liverpool Centre for Cardiovascular Science, Liverpool, UK; 4 Royal Brompton and Harefield Hospitals, Guy’s and St. Thomas’ NHS Foundation Trust, London, UK; 5 National Heart and Lung Institute, Imperial College London, London, UK

**Keywords:** 3D echocardiography, Ejection fraction, Left ventricle, Right ventricle, Survey, Chamber quantification

## Abstract

Three-dimensional echocardiography (3DE) imaging has permitted advancements in the quantification of left ventricular (LV) and right ventricular (RV) volumes and ejection fraction. We evaluated the availability of 3DE equipment / analysis software, the integration of 3DE assessment of the LV and RV in routine clinical practice, current training provisions in 3DE, and aimed to ascertain barriers preventing the routine use of 3DE for volumetric analysis. Through the British Society of Echocardiography (BSE) regional representatives’ network, echocardiographers were invited to participate in an open online survey. A total of 181 participants from echocardiography departments in the United Kingdom (UK), the majority from tertiary centres (61%), completed the 28-question survey. For 3DE quantification, 3DE-LV was adopted more frequently than 3DE-RV (48% vs 11%, respectively). Imaging feasibility was a recognised factor in 3DE RV and LV adoption. Many respondents had access to 3D probes (93%). The largest observed barriers to 3DE routine use were training deficiencies, with 83% reporting they would benefit from additional training opportunities and the duration of time permitted for the scan, with 68% of responders reporting allowances of less than the BSE standard of 45–60 min per patient (8% < 30-min). Furthermore, of those respondents who had undertaken professional accreditation, competence in 3DE was not formally assessed in 89%. This UK survey also reported good accessibility to magnetic resonance imaging (72%), which was related to overall 3DE adoption. In summary, although 3DE is now readily available, it remains underutilised. Further training opportunities, integrated formal assessment, improved adoption of BSE minimum recommended scanning times, alongside industry and societal support, may increase 3DE utilisation in routine practice.

**Supplementary Information:**

The online version contains supplementary material available at 10.1186/s44156-024-00044-1.

## Introduction

Quantification of left ventricular (LV) and right ventricular (RV) size and function is a key component to all transthoracic echocardiography (TTE) examinations [1, 2]. The introduction of three-dimensional echocardiography (3DE) has permitted advancements in the quantification of LV and RV volumes and ejection fraction (EF), all without the impact of geometric chamber assumptions, regional variations in contractility or passive cardiac motion [3, 4]. It is also the only echocardiographic modality allowing for direct measurement of RV volumes and EF without the need for surrogate linear markers of RV contractility. In addition, both 3DE derived LV and RV parameters have demonstrated superior prognostic importance compared to conventional two-dimensional echocardiography techniques [5–19], alongside published normal reference values [20, 21]. The British Society of Echocardiography (BSE) fundamentally supports best practice in the standardisation of echocardiographic image acquisition, analysis, and reporting to ensure highest diagnostic quality. As such, it recognises the superior reproducibility and added clinical and prognostic value of volumetric chamber assessment using 3DE [11, 22–24]. This is also reflected by both the European Association of Cardiovascular Imaging (EACVI) and the American Society of Echocardiography (ASE) guidance, whereby 3DE is favoured for volume and EF quantification for specific populations, such as cardiotoxicity screening [1, 25–27]*.*

Within the UK, even in the presence of growing scientific literature and societal guidance, echocardiography laboratories proficiency in 3DE adoption into clinical practice is anecdotally variable and the specific barriers are yet to be definitively understood. Consequently, the aims of this survey, with support from the BSE research and audit committee, were (i) to collect UK nationwide information from echocardiographers and clinicians performing TTE regarding their access to 3DE technology; (ii) to investigate the routine application of 3DE in the assessment of LV and RV volumes and EF compared to conventional and other novel quantitative indices across UK echocardiographic laboratories; and (iii) to identify the main barriers to wider implementation of 3DE in LV and RV quantification in routine clinical practice. These findings will assist in the development of measures aiming to overcome such barriers and enable support for greater 3DE adoption in routine every-day clinical practice to drive the most accurate and efficient echocardiographic services for our patients.

## Methods

The survey was designed for all healthcare professionals performing TTE studies in the UK and was conducted during six weeks from June 5th to July 16th, 2023. It was primarily advertised through the BSE regional representative network, BSE website, and BSE social media channels. Participation in the survey was through an open access weblink (SurveyMonkey) to a structured questionnaire comprising a total of 28 questions. The questions covered several areas including current approach to routine LV/RV echocardiographic assessment and reporting, access to 3DE technology and specific training, professionals’ views on the value and feasibility of 3DE derived volumetric analysis, and availability of alternative imaging modalities at their place of work. The full questionnaire is available in Additional file 1. Most questions required binary yes/no answers or had multiple choices, but on occasion free text answers were viable, allowing for collection of more detailed explanations. There were two reminders sent after the second and fourth weeks (19th of June and 3rd of July 2023). All answers were treated in strict confidence and fully anonymised. All responders provided informed consent for participation in the survey. The survey registration was approved by the Liverpool Heart and Chest Hospital ethical committee (SR100017).

## Results

**Fig. 1:**
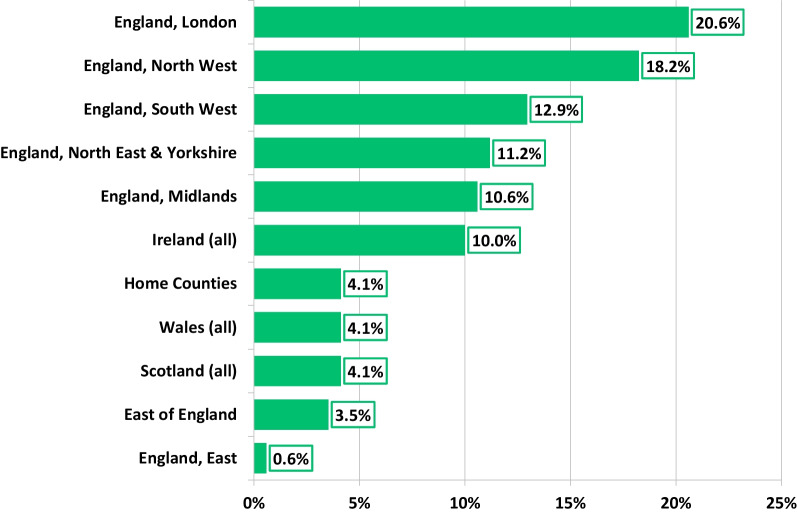
Geographic location and percentage of respondents

**Fig. 2:**
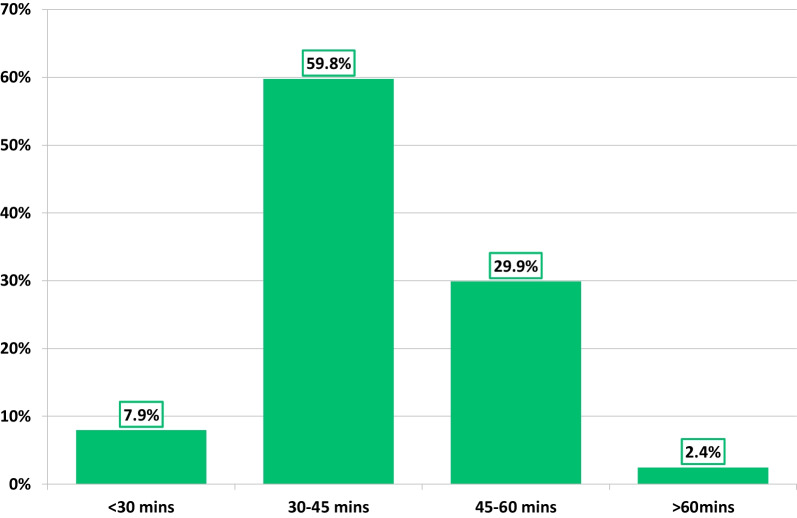
Time allocated to scan and report a TTE exam

One hundred and eighty-one participants responded to the survey, with 156 completing all questions. Response percentages varied from 100 to 86%. There was a nationwide response (Fig. 1), the majority largely practised in an NHS tertiary centre (61%), followed by district general hospital (32%), community care setting (4%), or a private hospital (3%). Overall, 88% of respondents were based in adult echocardiography departments (acquired and/or congenital), of which, only 54% held departmental accreditation by either BSE of EACVI. National or international accreditation in TTE was held by 95% of the respondents, of which, 89% were BSE level 2 accredited. With respect to departmental scan time provision for a standard TTE slot (scan and report), only 32% reported in line with the BSE recommendation of 45–60 min or more (Fig. 2).

### Access to 3DE technology

Ninety three percent of respondents had access to 3D TTE probes in their department, with 31% reporting full availability (i.e., all their machines), 40% reported they were largely available (> 50% of machines), and 22% reported they were rarely available (< 50% of machines). The respondents reported that 73% had access to 3DE-LV software on cart. When asked about the availability of 3DE-RV software, 43% had availability on cart, 15% offline, with the remaining not having access to the software package (21%) or unknown (21%). Both cardiac magnetic resonance (CMR) and computed tomography (CT) cross-sectional imaging modalities were widely available (72% and 83%, respectively) at the respondent’s centres.

### Left ventricle

#### Role and application of 3DE in the assessment of LV volumes and EF

**Fig. 3:**
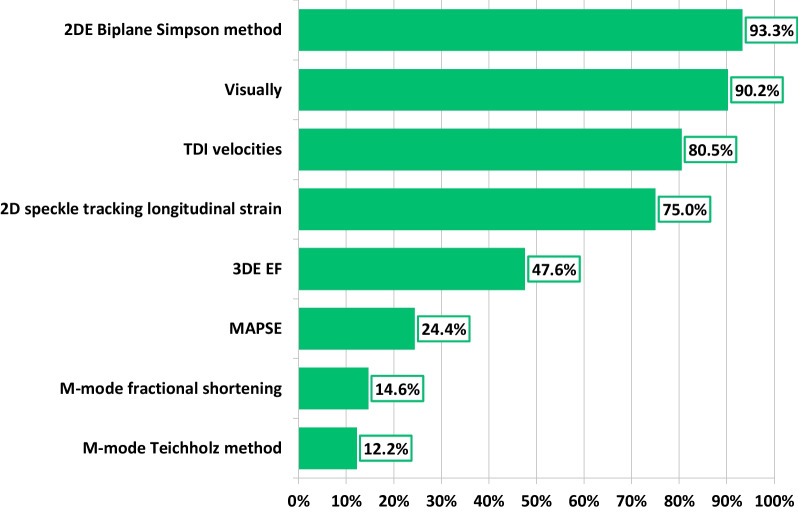
Methods of assessing LV function in routine TTE

**Fig. 4:**
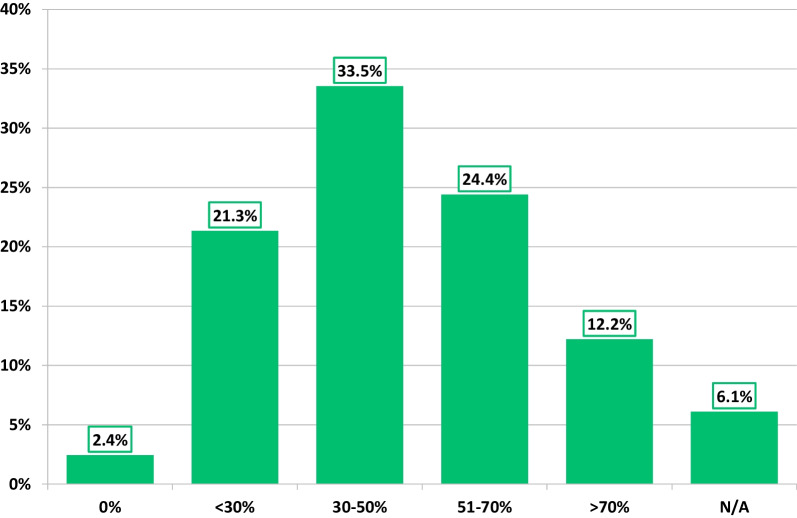
Proportion of respondents able to acquire 3DE LV full volume datasets in 0%, < 30%, 30–50%, 51–70% and > 70% of their patients

Most respondents (91%) agreed that accurate assessment of LV volumes and EF were imperative to their echocardiographic examination. While the most frequently used TTE method for routine quantification of LV EF was Biplane Simpson’s method (93%), assessment of LV function by speckle tracking global longitudinal strain (GLS) and 3DE LV EF were routinely performed by 75% and 48% respondents respectively (Fig. 3). Although only 32% reported that 3DE-LV EF quantification was part of their departmental standard TTE protocol, 68% felt positive about its adoption, with 62% believing it should now be introduced into routine TTE protocols. Currently, 36% of respondents were generally able to acquire 3DE full volume datasets of sufficient quality for LV volumetric analysis in > 50% of their patients (Fig. 4). Reporting of LV function based solely on two parameters, EF and GLS, has become current practice for over half (56%) of respondents, with a further 20% agreeing, but do not currently report this way.

#### Training in 3DE LV quantification

Most respondents reported previous exposure to training in using 3DE TTE to assess LV EF (62% by the manufacturer, 41% by department colleagues, 13% at conference sessions, 13% at a dedicated training course), but 83% of respondents reported they would like further training. Although 70% of respondents had colleagues within their department with experience in 3DE-LV assessment that they could approach for advice, most (86%) reported that their accreditation did not incorporate any theoretical or practical examination on 3DE.

#### Potential barriers to wider implementation of 3DE in LV quantification

Besides challenges related to the lack of training and acquisition of good quality 3DE datasets being most frequently cited as the main barriers to the adoption of 3DE-LV assessment (48%), other barriers included: too time consuming (45%), over complication in the analysis process (24%), and lack of confidence in the values produced (24%). When asked for personal comments regarding reasons for not performing 3DE-LV assessment, common themes included lack of knowledge in reference values for 3DE derived volumes and EF, conservatism, and test layering (i.e., “*good 3D, don’t do biplane*”), lack of offline / off-cart analysis system availability and the notion of “*wasted effort*” by the referring clinical colleagues due to reliance on standard TTE parameters or CMR. Many appreciated CMR being the gold standard for accurate LV volumetric and EF assessment, specifically in patients with poor acoustic window, large body mass index and/or complex congenital heart disease.

### Right ventricle

#### Role and application of 3DE in the assessment of RV volumes and EF

**Fig. 5:**
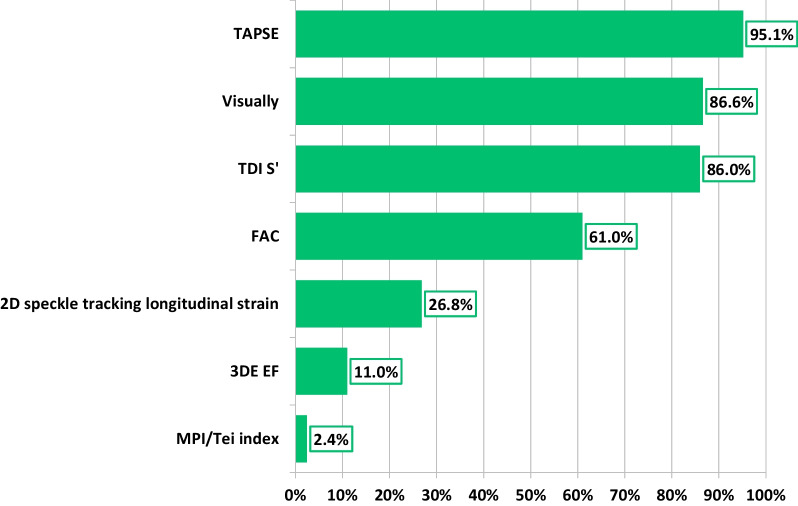
Methods of assessing RV function in routine TTE

**Fig. 6:**
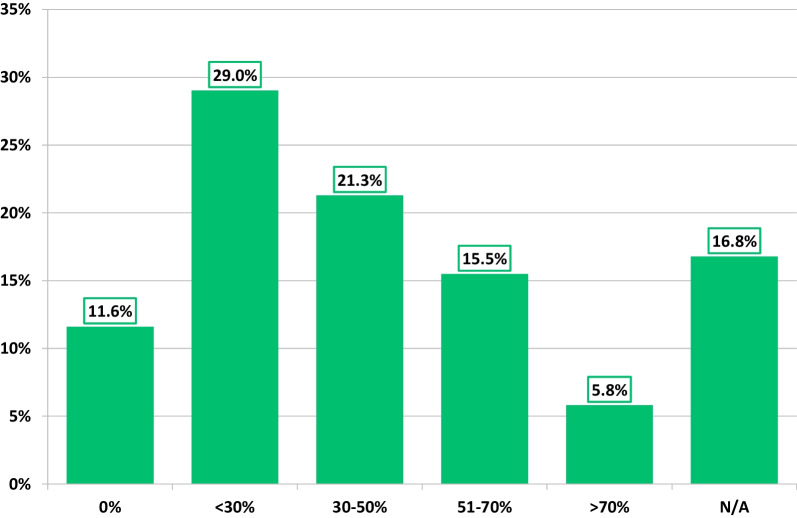
Proportion of respondents able to acquire 3DE RV full volume datasets in 0%, < 30%, 30–50%, 51–70% and > 70% of their patients

Most respondents (87%) agreed that accurate assessment of RV size and EF was imperative to their echocardiographic examination. While the most frequently used parameter of RV systolic function was TAPSE (95%), RV function assessment by speckle tracking and 3DE-RV EF was routinely performed by 27% and 11% of respondents, respectively (Fig. 5). Only 10% reported 3DE-RV EF as being part of their standard departmental TTE protocol. While 43% felt positive about 3DE-RV routine adoption, others were either uncertain (41%) or believed further software development is needed prior to being introduced into routine practice (16%). On average, 50% of respondents were not generally able to acquire 3DE datasets of sufficient quality for RV volumetric analysis (Fig. 6). Although some respondents agreed that the assessment of RV function should solely be based on two parameters only—EF and strain (26%), the majority do not currently report this way, including 32% who actively disagreed.

#### Training in 3DE RV quantification

Over half of the respondents (53%) reported no exposure to any dedicated 3DE-RV training. Others received some training provided by the manufacturer (34%), by department colleagues (24%), at conference sessions (7%), and 6% attended a dedicated training course. Interestingly, although 47% had colleagues with exposure in 3DE-RV assessment they could approach for advice, 40% were unable to receive any guidance. A vast majority (83%) of respondents would like to receive further 3DE-RV training.

#### Potential barriers to wider implementation of 3DE in RV quantification

Analogous to 3DE-LV assessment barriers, the most common obstacles for 3DE-RV assessment included: patient feasibility (46%), time consumption in acquisition and analysis (36% and 23%, respectively), lack of competency or confidence (32% / 27%) and a perception of lack of reproducibility (27%). When asked for personal comments regarding reasons for not performing 3DE-RV, in addition to themes mirroring 3DE LV assessment, commentary included challenging visualisation of the RV anterior wall, unreliable algorithms for pathologies where RV geometry is altered (i.e., congenital heart disease), “*limited validated data to break into clinical guidelines*”, lack of clinical demand and reliance on CMR*.*

## Discussion

To our knowledge this is the first UK survey to provide insight from a range of echocardiographic laboratories and healthcare settings on the use and adoption of 3DE for LV and RV evaluation. In line with other international echocardiographic communities, this data demonstrated that 3DE employment remains variable and generally underutilised compared to conventional echocardiographic indices. Given our data is reflective of largely tertiary centre respondents with over half being departmentally accredited, it is conceivable that the overall adoption and availability of 3DE across all UK echocardiography departments is in fact lower than our results suggest. This is since it is more likely that tertiary centres and/or accredited departments will have the requirement, training, and resources for implementing 3DE compared to secondary or primary care environments or non-accredited departments. Reservation around 3DE adoption was largely ascertained to a persistent uncertainty in the additive value of the 3DE volumetric approach, particularly of the RV [7]. This is despite the growing body of evidence demonstrating superior prognostic benefits and better reproducibility of 3DE of the LV and RV when compared to conventional TTE parameters [5–19]. The survey findings highlighted imaging feasibility, time constraint, and training deficiencies as the key contributing barriers to routine adoption. Additionally, although it is evident that most UK echocardiographic laboratories now have 3DE equipment, access to 3DE analysis software is still relatively limited, especially for the RV.

### Current place of 3DE in assessment of the LV and potential barriers to its wider use

A third of respondents reported that 3DE derived LV quantification was part of their standard TTE protocol with a majority believing it should now be part of standard practice within UK echocardiography laboratories. However, limitation still exists at end-user level. The frequently reported lack in ability to acquire and analyse 3DE-LV datasets reliably cannot be fully attributed to poor imaging quality or complicated post-processing/“*user-friendliness of the navigation platform*” and is likely to reflect a need for continued training in 3DE-LV data acquisition and analysis (as being desired by 83% of responders). Given the reported reasonable availability of dedicated 3DE-LV software, it is perhaps a component that should now be considered more formally in a TTE accreditation or sub-accreditation pathway specifically for 3DE. As 3DE is a relatively novel imaging modality of continued evolution, it is unsurprising that although most respondents held TTE accreditation, 3DE was not often formally part of the respondent’s accreditation criteria. Future 3DE learning/teaching pathways, such as a societal endorsed eLearning module and/or teaching and training events would help “fill the 3DE knowledge and experience gap”, support continued professional development and ultimately combat some of the highlighted 3DE barriers. Further It was also highlighted that a potential barrier to 3DE-LV assessment was the lack of contemporaneous normative reference values for 3DE-LV volumes and EF breaking into societal guidelines [20].

Although LV EF is a robust diagnostic and prognostic marker relied upon in various cardiovascular diseases [5], the measurement parameter itself is not without its own limitations with respect to reliably quantifying cardiac function. This applies to all imaging modalities with shortcomings largely reflecting the potential consequential impacts of haemodynamic, electrophysiological, and valvular function status, as well as the effects of certain cardiac procedures (i.e., VSD patch). Interestingly, TDI S’ was utilised by 80% of respondents, yet this is not a primary quantification parameter in societal recommendations for chamber quantification [1, 2]. This observation is likely to reflect the high feasibility of the measurement.

### Current place of 3DE in assessment of the RV and potential barriers to its wider use

Most respondents agreed that RV function assessment is fundamental. Although visual assessment and conventional parameters are easy, fast, guideline recommended [1, 22, 23], they are fundamentally problematic in that they have limited representation of global RV function. Nonetheless, they remain reported much more frequently compared to 3DE and RV speckle tracking longitudinal strain. This can serve as both an explanation and the consequence as to why more than half of respondents had never received dedicated training in 3DE-RV assessment, equally why many did not have a colleague within their department with any experience within 3DE-RV, and inevitably why a low ability to acquire suitable 3DE-RV datasets was reported. This is analogous with the recent World Alliance Societies of Echocardiography 3DE-RV reference data findings [21]. It is therefore unsurprising why only 10% of respondents reported that their department utilise 3DE-RV within their routine departmental TTE protocol.

The underutilisation of 3DE-RV is also likely reflective of the technical challenges in acquiring sufficient quality quantifiable datasets. Unlike 3DE-LV, the complexities of RV morphology result in difficulties when visualising larger ventricles and in particular the anterior wall [27, 28]. The survey overall highlighted that further training in 3DE-RV acquisition and analysis and better access to dedicated software packages are required. However, akin to 3DE-LV, a lack of contemporaneous normative reference values for 3DE-RV volumes and EF breaking into societal guidelines was again highlighted as a potential barrier [21]. Interestingly, societal guidelines recommend that global RV function should be evaluated [1, 22, 23], and this remains largely carried out through the application of RV FAC% over 3DE-RV, which was indicated in our UK survey data and likely reflects the BSE minimal dataset requirements [3].

### Common barriers of 3DE wider use

Time constraint was reported as an important obstacle to overall 3DE utilisation. Given only 32% of respondents reported a standard TTE slot time (scan and report) in line with the BSE recommendation of 45–60 min, with a further 8% reporting < 30-min, it is clear why more advanced practices such as 3DE and speckle tracking are not being routinely adopted. Even with more novel automated processing technologies that have demonstrated accurate, fast, and reproducible 3DE chamber analysis, impactful improvement in overall TTE study times compared with conventional TTE remains to be demonstrated [29]. It is additionally anticipated that TTE examination timings are and will continue to be balanced against meeting the overwhelming service demands and current UK backlog waiting lists alongside a diminished workforce. This is unfortunately irrespective of the recently published BSE triaging platform documentation [30]. Perhaps one may postulate a shift in mindset to a notion of “*measurement conservatism and test layering*” to help in easing TTE time constraints, for example, good 3DE datasets could negate repetition in LV and RV size and function measurements by other conventional TTE indices all together. The survey also reflected a level of uncertainty in the additive value of 3DE, with many respondents portraying a substantial reliance on CMR, because it is “gold standard” and owing to its easy accessibility within the UK. It is important to ensure thorough understanding of the benefits and limitations of 3DE and CMR. For example, 3DE has advantageous utilisation in those patients that are contraindicated (i.e., pacing leads), not amenable (i.e., claustrophobia), and/or those with known significant artefact issues (i.e., metal). Environmental aspects of cardiovascular imaging with dramatically higher carbon emissions produced by CMR compared to TTE should also be contemplated [31].

### Future perspectives

Assessment of LV and RV size and function by 3DE imaging methods is a continually evolving field. The use of 3DE can overcome some of the limitations of standard TTE. Further improvement in 3DE frame rate acquisition for improved measurement reproducibility, particularly for 3DE-RV, faster processing algorithms, alongside the potential for inter-vender standardisation will undoubtedly support 3DE expansion into routine practice and wider clinical implementation. The introduction of 3DE strain imaging is a promising technology and is emerging as a more physiologically sound tool for analysing the complexities of LV myofiber contractile architectural mechanics [16, 32–34]. Further development and research are needed and should be encouraged for future introduction.

### Limitations

We acknowledge that this data is derived from an online survey and therefore has related limitations. Data was collected anonymously; thus, the accuracy of the individual responses cannot be verified. Although respondent data is reflective of a real-world UK cohort, the overall number of survey respondents is relatively low when considering the number of UK professionals performing TTE, with ~ 4500 BSE members, and consequently does lack comprehensive UK TTE workforce representation The survey was voluntary which may reflect respondent bias. Responses may also be bias based on the potential interpretations of the questions. Finally, given many respondents worked in either a tertiary or district general hospital environment, it may lack generalisability to other clinical environments, such as community care settings.

## Conclusion

There is clear evidence that now supports the adoption of 3DE for LV and RV quantification in clinical practice, however it remains underutilised across UK centres. The survey demonstrates a need for education around 3DE, on its benefits, pitfalls, normative reference values and understandings in which patient groups 3DE would particularly be beneficial. Extensive training alongside ongoing user-friendly evolution in 3DE technology is imperative and echocardiography societies and industry partners are encouraged to promote and provide the tools and educational materials to drive this. With continued and collaborative support from echocardiographic communities, 3DE should become more widely and easily adopted within everyday echocardiographic clinical practices. This will ensure the continuation of best patient care provision.

### Supplementary Information

**Additional file 1: **The full questionnaire.

## Data Availability

Full questionnaire is available in Additional file 1: Table S1. The data underlying this article will be shared on reasonable request to the corresponding author.
